# Electrochemical Characterization and Sensitive Voltammetric Determination of Etamsylate at a Boron-Doped Diamond Electrode

**DOI:** 10.3390/mi17030299

**Published:** 2026-02-27

**Authors:** Joanna Smajdor-Baran, Katarzyna Fendrych, Bogusław Baś, Robert Piech

**Affiliations:** Department of Analytical Chemistry and Biochemistry, Faculty of Materials Science and Ceramics, AGH University of Krakow, 30-059 Krakow, Poland

**Keywords:** etamsylate, Boron-Doped Diamond electrode, Square Wave Voltammetry

## Abstract

This study presents the development of a highly sensitive and selective electrochemical method for the determination of etamsylate (ETM) using a Boron-Doped Diamond (BDD) electrode. Analytical conditions were optimized using Square Wave Voltammetry (SWV), establishing an orthophosphoric acid (V) solution as the ideal supporting electrolyte. Under optimized instrumental parameters, the sensor demonstrated a superior linear response with a correlation coefficient of 0.999. The calculated limit of detection (LOD) was 0.10 µmol L^−1^ (0.026 mg L^−1^), with very good repeatability, indicated by an RSD of 3.17%. The practical utility of the BDD electrode was confirmed through the analysis of pharmaceutical and spiked biological samples, yielding recovery values between 95% and 102%. The results indicate that this method serves as a robust, cost-effective, and efficient alternative for routine quality control, clinical diagnostics, and environmental monitoring of etamsylate.

## 1. Introduction

Etamsylate (ETM), chemically known as 2,5-dihydroxy-benzene-sulfonate diethylammonium salt, is a synthetic, water-soluble non-steroidal hemostatic agent that has been widely used in clinical practice for the prevention and treatment of capillary bleeding [1,2,3]. Unlike classical coagulation-modifying drugs, etamsylate primarily acts at the level of the microvasculature, improving capillary resistance and reducing abnormal blood loss without significantly affecting systemic coagulation parameters [3]. Clinically, etamsylate has been applied across various medical disciplines, including gynecology, obstetrics, surgery, otorhinolaryngology, and ophthalmology, for the management of capillary bleeding of diverse etiology [4,5,6,7,8,9]. The agent has demonstrated efficacy in reducing intra- and postoperative hemorrhage, and is also utilized in specific settings such as the prevention of periventricular hemorrhage in preterm neonates and the control of diffuse capillary bleeding in surgical patients [2].

Owing to its widespread therapeutic application, reliable and accurate analytical methods for the qualitative and quantitative determination of etamsylate in bulk materials, pharmaceutical dosage forms, and biological matrices are essential for quality control, regulatory compliance, and pharmacokinetic studies. From an analytical chemistry perspective, etamsylate presents several challenges related to its physicochemical properties, including high polarity, strong hydrophilicity, and the presence of phenolic and sulfonate functional groups. Consequently, the development of robust analytical methodologies requires careful selection of separation techniques, detection modes, and sample preparation procedures.

Several analytical methods have been developed for the quantitative analysis of etamsylate in pharmaceutical and biological matrices, including most of all high-performance liquid chromatography (HPLC) [10,11,12] and spectrophotometric methodologies [13,14,15,16]. Nevertheless, the high polarity and pronounced hydrophilicity of etamsylate often result in insufficient retention on conventional reversed-phase stationary phases, leading to poor peak shape, early elution, and reduced separation efficiency. To overcome these drawbacks, method development frequently requires the use of ion-pairing reagents, highly aqueous mobile phases, or alternative stationary phases, which may compromise method robustness, reproducibility, and column longevity. While spectrophotometric techniques provide a convenient and inexpensive means for etamsylate quantification, they are often limited by insufficient selectivity and interference from coexisting compounds, which can compromise analytical accuracy and necessitate additional sample preparation or chemometric correction. Taken together, these limitations highlight the need for alternative or complementary analytical strategies that offer simplified sample preparation, reduced solvent usage, and improved suitability for the determination of etamsylate in both pharmaceutical formulations and biological matrices, while maintaining adequate sensitivity, selectivity, and reproducibility.

Electrochemical techniques, particularly voltammetry, are highly suitable for overcoming these analytical challenges, providing both high sensitivity and selectivity while minimizing sample preparation steps and solvent consumption [17]. The electroactive phenolic moieties of etamsylate facilitate direct electron transfer at the electrode surface, enabling rapid, accurate, and reproducible quantification. To date, voltammetric determination of etamsylate has been carried out on a 4-amino-2-mercaptopyrimidine self-assembled monolayer gold electrode [18], a glassy carbon electrode modified by hydrophobic gold nanoparticles [19], and a multi-walled carbon nanotube-modified glassy carbon electrode [20].

However, Boron-Doped Diamond electrodes (BDDEs), despite their well-documented advantages in terms of wide potential window, low background current, chemical inertness, and fouling resistance [21,22], have never been applied for the electrochemical analysis of etamsylate. These properties not only permit repeated measurements without significant loss of sensitivity but also enhance the signal-to-noise ratio, allowing reliable detection of etamsylate even at trace concentrations. Taken together, these characteristics make BDDE-based voltammetric methods a promising platform for the development of simple, robust, and environmentally sustainable analytical procedures for both pharmaceutical formulations and complex biological matrices [23,24,25].

This work presents a comprehensive electrochemical investigation of etamsylate, focusing on both its redox mechanism and sensitive quantitative determination. Linear sweep voltammetry (LSV) was employed to elucidate the kinetic parameters of the oxidation process. Furthermore, Square Wave Voltammetry (SWV) was utilized for the electroanalytical quantification of the drug. SWV was chosen for quantitative analysis due to its superior sensitivity and effective discrimination against capacitive currents compared to LSV. The combination of mechanistic insights and optimized SWV parameters provides a robust and sensitive platform for the monitoring of etamsylate levels in complex matrices.

## 2. Materials and Methods

### 2.1. Chemicals and Solutions

The primary analyte, etamsylate (ETM, purity ≥ 95%), was sourced from Sigma Aldrich (St. Louis, MO, USA). All auxiliary chemicals were of analytical grade and utilized as received. A 0.01 mol L^−1^ primary stock of ETM was prepared in deionized water and kept refrigerated (4 °C); subsequent working dilutions were freshly made each day. Orthophosphoric acid (≥85%) was also provided by Sigma Aldrich. For validation purposes, the study utilized commercial synthetic human plasma (Biowest, Nuaillé, France) and certified wastewater reference materials (SPS-WW1/SPS-WW2 from Spectrapure Standards).

### 2.2. Instrumentation

Electrochemical data were acquired using an mtm-anko (Kraków, Poland) M161 analyser coupled with an M164 stand. Software control, including signal recording and subsequent analysis, was performed via EALab (v. 2.1) and Origin 2023b. A classical three-electrode cell was employed, featuring a 3 mm Boron-Doped Diamond working electrode with PEEK body (M-BDD-3, OD: 7 mm, ID: 3 mm, BioLogic, Seyssinet-Pariset, France), a Ag/AgCl reference (3 mol L^−1^ KCl, Mineral, Łomianki, Poland), and a platinum auxiliary electrode. The BDDE boron doping level was between 500 and 1000 ppm and the average roughness parameter (R_a_) of approximately < 10 nm (manufacturers declarations).

### 2.3. Voltammetric Procedures

The electrochemical behaviour of etamsylate was explored using Cyclic Voltammetry (CV) and Square Wave Voltammetry (SWV), the latter being optimized for quantitative determination. Experiments were conducted in a 0.1 mol L^−1^ H_3_PO_4_ supporting electrolyte, scanning from 0 to 1500 mV. The refined SWV conditions included a frequency (*f*) of 50 Hz, a pulse amplitude (*dE*) of 50 mV, and a step potential (*E_s_*) of 5 mV, with both sampling and waiting times (*t_p_*, *t_w_*) set to 5 ms. Kinetic studies involved varying the CV scan rates within the range of 6.3–500 mV s^−1^.

### 2.4. Sample Preparation

#### 2.4.1. Tablets

For the quantitative etamsylate measurements in the pharmaceutical formulations, a prescription drug was purchased from the local pharmacy. To ensure uniformity, three tablets were pulverized in a mortar. An accurately weighed portion of the resulting powder was dissolved in deionized water within a volumetric flask. Prior to analysis, the mixture was passed through a 0.45 µm regenerated cellulose (RC) syringe filter (Biosens, Warsaw, Poland) and diluted as required in Eppendorf tubes.

#### 2.4.2. Plasma

Plasma samples (Biowest, France) were kept at −20 °C until use. To eliminate protein-based interferences, a deproteinization step was performed: 800 μL of plasma was treated with 200 μL of 10% trichloroacetic acid (TCA, Sigma Aldrich, USA). The mixture was vortexed (Biosan, Nuaillé, Poland) for 2 min and then centrifuged at 10,000 rpm for 30 min (Eppendorf, Hamburg, Germany). The resulting clear supernatant was recovered, filtered through a 0.45 μm RC membrane, and then subjected to voltammetric analysis.

#### 2.4.3. Wastewater CRM

The wastewater CRM samples required minimal handling. They were introduced directly into the electrochemical cell following a 5-fold dilution with double-distilled water.

### 2.5. Real Samples Analysis

To ensure the accuracy of ETM determination in samples with complex matrix such as tablets, plasma or wastewater, a combined dilution-standard addition method approach was employed to overcome selectivity challenges. Each sample was diluted x-fold (10×, 33× and 20×) with the supporting electrolyte of 0.1 mol L^−1^ orthophosphoric acid. This step is essential in voltammetry to minimize electrode fouling caused by the adsorption of matrix macromolecules, bring the concentration of interferents below their interference threshold and stabilize the double-layer capacity, ensuring a reproducible background current. After the initial dilution, a series of four subsequent voltammetric scans were performed. Following the measurement of the sample, three successive increments of a standard solution were added directly into the electrochemical cell. The peak current and potential was monitored after each addition. The unknown concentration of ETM in the original sample was determined using the standard addition plot method. The peak currents were plotted against the added concentrations of the standard, and the intercept on the concentration axis provided the analyte concentration in the measuring cell. To find the final concentration in the initial sample, the result was adjusted by the dilution factor.

## 3. Results and Discussion

### 3.1. Influence of Supporting Electrolyte Composition on Etamsylate Peak

The electrochemical response of etamsylate was evaluated across a diverse range of supporting electrolytes to determine the most suitable medium for analysis (Figure not included). The tested solutions included 0.035 mol L^−1^ acetic acid, 0.1 mol L^−1^ acetate buffer (pH 3.0), 0.1 mol L^−1^ orthophosphoric acid (V), 0.1 mol L^−1^ sulfuric acid (VI), 0.05 mol L^−1^ perchloric acid, 0.1 mol L^−1^ phosphate buffer (pH 7.0), and 0.1 mol L^−1^ borate buffer (pH 9.3). Analysis of the resulting voltammograms indicated that 0.1 mol L^−1^ orthophosphoric acid (V) provided the most favourable analytical conditions. While other media, particularly those with higher alkalinity or extreme acidity, resulted in diminished peak currents or the complete absence of the signal, the orthophosphoric acid medium yielded a well-defined oxidation peak with a significantly reduced background current. Consequently, 0.1 mol L^−1^ orthophosphoric acid was selected as the optimal supporting electrolyte for all subsequent quantitative determinations of etamsylate.

### 3.2. Influence of the SWV Parameters on Etamsylate Peak

To ensure the maximum highest sensitivity for the detection of etamsylate with the BDDE, the Square Wave Voltammetry (SWV) parameters underwent a rigorous optimization process. A comprehensive evaluation of various operational factors was conducted, specifically targeting the potentials of the pulse and step potential, the frequency, and the durations for both sampling and waiting. The refinement of these variables led to the identification of an optimal instrumental setup, characterized by a pulse potential (*dE*) of 50 mV and a step potential (*E_s_*) of 5 mV. Furthermore, the frequency (*f*) was set to 50 Hz, with both the sampling (*t_p_*) and waiting (*t_w_*) times fixed at 5 ms. Notably, the accumulation time was excluded from the optimization parameters, as the analyte showed no evidence of pre-concentration on the BDDE surface. These calibrated settings were subsequently utilized as the baseline for all further experimental data collection.

### 3.3. Voltammetric Behaviour of Etamsylate on the BDD Electrode

**Figure 1:**
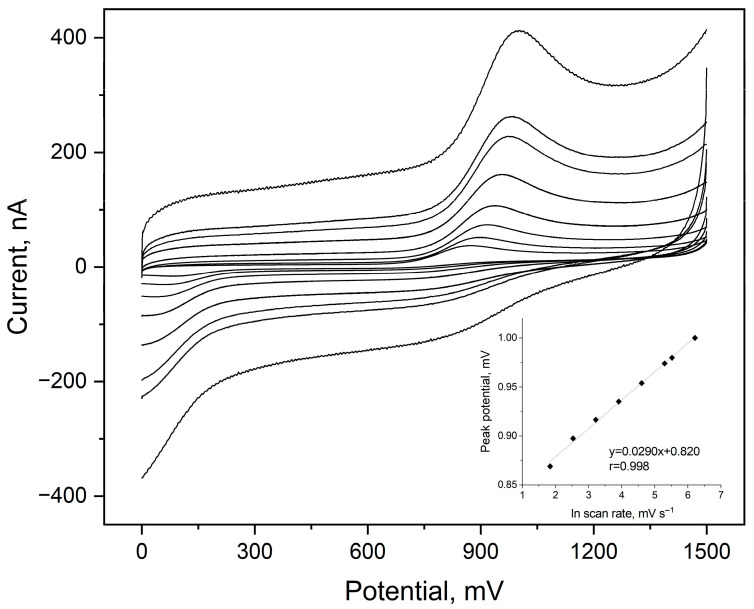
Cyclic voltammograms registered for 5 µmol L^−1^ of etamsylate in a 0.1 mol L^−1^ orthophosphate acid in a scan rate range from 6.3 to 500 mV s^−1^ ETM peak visible in the anodic scans. Dependences of the ETM peak potential on the natural logarithm of scan rate values as the inset of the plot.

The influence of scan rate (*ν*) on the electrochemical oxidation of etamsylate was evaluated using Cyclic Voltammetry across a range of 6.3 to 500 mV s^−1^. The analysis was performed to determine the nature of the mass transport and the electron–proton stoichiometry of the redox reaction. A strong linear correlation was observed between the anodic peak current (*I_pa_*) and the square root of the scan rate (*ν*^1/2^), as evidenced by the high determination coefficient (R^2^ = 0.999). The linearity of this relationship confirms that the oxidation of etamsylate at the BDDE surface is a diffusion-controlled process. The diffusion-controlled nature suggests that the mass transport of etamsylate from the bulk solution to the electrode surface is the rate-limiting step in the overall electrochemical process. Furthermore, the anodic peak potential (*E_pa_*) shifted toward more positive values as the scan rate increased, a characteristic behaviour of an irreversible electrochemical process (Figure 1).

To further characterize the electrode process, of etamsylate oxidation on BDDE, the number of electrons taking part in this reaction was calculated. The peak potential (*Ep*) shifted towards more positive values with increasing scan rate, confirming the irreversible nature of the electron transfer. The plot of *Ep* versus ln*v* yielded a straight line with a slope of 0.029 V. According to the theory of irreversible processes, the slope of this dependence can be described using following equation:

(1) $$s=\frac{RT}{2\alpha nF}$$

Assuming a standard transfer coefficient (α) of 0.5 for a totally irreversible system, the temperature (*T*) value of 298 K, universal gas constant (*R*) as 8.314 J mol^−1^ K^−1^ and the Faraday constant (*F*) as 96,485 C mol^−1^, the number of exchanged electrons was calculated as 1. To confirm the calculation, the following equation was also implemented

(2) nα=0.048Ep−Ep12

The calculated nα value was equal to 0.46, which considering α as typical 0.5, gives the result of one electron exchanged in the ETM oxidation reaction on the BDDE.

**Figure 2:**
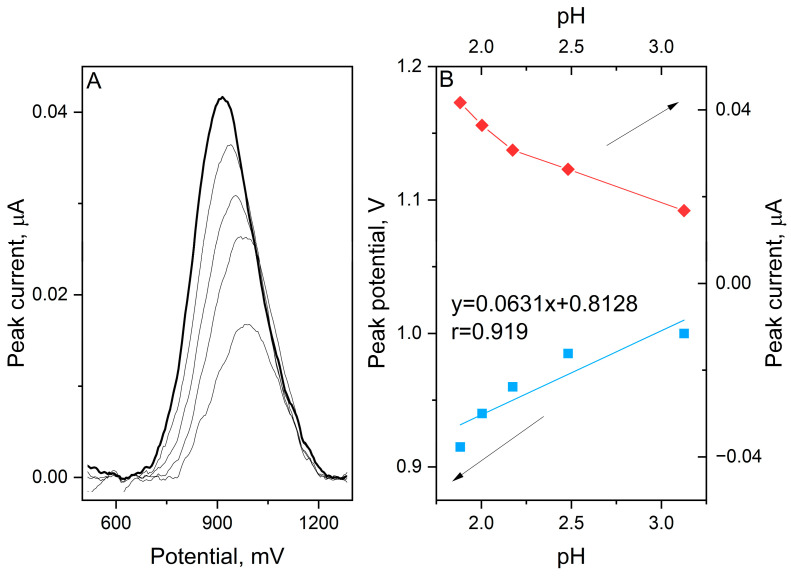
SWV voltammograms registered for oxidation of 1 μmol L^−1^ etamsylate in 0.1 mol L^−1^ orthophosphoric acid with pH values ranging from 1.9 to 3.1 (**A**). The dependence between the peak current and potential on the pH of the supporting electrolyte (**B**).

Furthermore, the impact of the electrolyte acidity on the electrochemical response was investigated within a pH range of 1.88 to 3.17 (Figure 2).

Interestingly, a positive correlation between the anodic peak potential and pH was observed. The *E_pa_* shifted toward more positive values as the pH increased, following the linear equation:

(3) $$E_{p}=0.063pH+0.813V$$

The calculated slope of 63.1 mV/pH is very close to the theoretical Nernstian value of 59.2 mV/pH, which suggests that the number of electrons and protons involved in the rate-determining step is equal. Typically, organic oxidations exhibit a decrease in potential with increasing pH due to deprotonation. However, the observed positive slope in this study points toward a specific reaction pathway. This behaviour suggests that the oxidation of etamsylate is preceded by a protonation step which implies a chemical–electrochemical mechanism of oxidation reaction. In this framework, the protonated form of the molecule is the electroactive species. As the pH increases and the concentration of H^+^ ions decreases, the preceding protonation becomes thermodynamically less favourable, thus requiring a higher overpotential to drive the reaction.

**Scheme 1:**
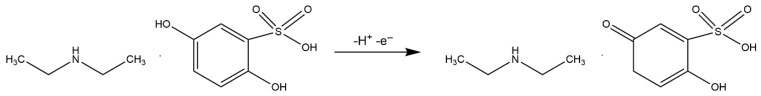
Possible mechanism of etamsylate oxidation reaction on the BDD electrode.

Correlating the results from the scan rate studies and the pH dependence, it can be concluded that the electrochemical oxidation of etamsylate at the BDDE surface involves the transfer of one electron and one proton (1e^−^/1H^+^), with a crucial role played by the pre-protonation of the analyte in acidic media. The proposition of the possible oxidation reaction mechanism is presented in Scheme 1.

### 3.4. Interference Studies

**Figure 3:**
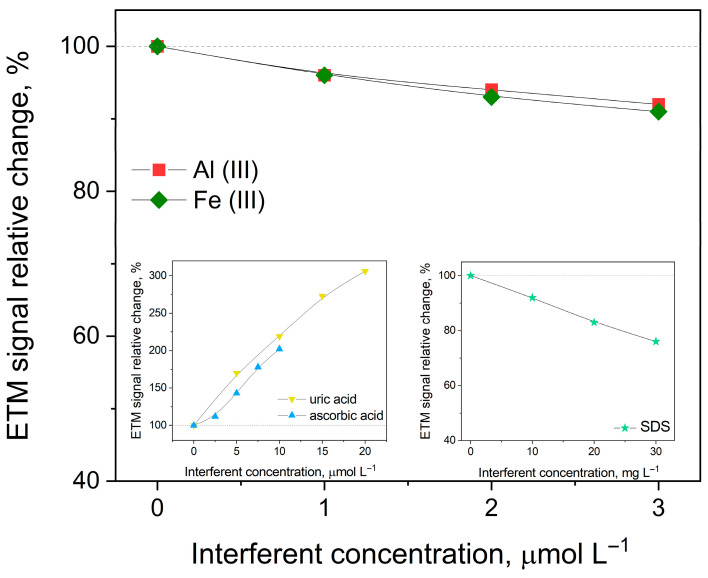
Dependence of the peak current on selected interferents on 1 µmol L^−1^ ETM concentration in 0.1 mol L^−1^ orthophosphoric acid. Other instrumental parameters as in point 2.3.

To evaluate the selectivity of the developed method for etamsylate determination, the influence of various potential interferents—both inorganic ions and organic compounds—was systematically investigated and evaluated by monitoring changes in the peak current (*I_p_*) and peak potential (*E_p_*) of etamsylate. The impact of common metal ions was assessed by adding 3 μmol L^−1^ of Fe(III), Zn(II), Al(III), Mo(VI), Pb(II), Co(II) and Cu(II), as well as higher concentrations (1 mmol L^−1^) of K, Mg(II) and Ca(II) to the analyzed solutions. Furthermore, the study extended to organic molecules typically found in biological fluids or pharmaceutical formulations. These included glucose and saccharose (0.1 mmol L^−1^), lactose monohydrate (60 μmol L^−1^), ascorbic acid (10 μmol L^−1^), and uric acid, alongside caffeine and aspartame (20 μmol L^−1^). The potential effects of common excipients, such as microcrystalline cellulose and magnesium stearate (20 μmol L^−1^), were also examined. Finally, the influence of surface-active agents was tested using the non-ionic surfactant Triton X-100 (0.3 μg mL^−1^), as well as the ionic surfactants sodium dodecyl sulfate (SDS) and cetyltrimethylammonium bromide (CTAB) (30 mg L^−1^). Significant deviations were observed for several specific ions and molecules. The presence of Al(III) and Fe(III) resulted in a moderate suppression of the analyte signal, with current decreases of 8% and 9%, respectively. Additionally, Fe(III) caused a positive shift in the peak potential of 20 mV. A more pronounced potential shift was noted for Cu(II), which displaced the etamsylate peak by 30 mV toward more positive values. The most substantial impacts were observed with biologically active compounds. The addition of ascorbic acid led to a two-fold increase in the etamsylate signal, accompanied by a 25 mV anodic shift in peak potential. Uric acid exhibited the most significant interference, resulting in a three-fold enhancement of the ETM peak current and a substantial peak displacement of 100 mV toward higher potentials. Also, the ionic surfactant SDS noticeably hindered the electrochemical process, causing a 24% reduction in the peak current and shifting the peak potential by 35 mV. The observed signal enhancements and potential shifts, particularly in the presence of ascorbic acid, uric acid, and SDS, indicate significant matrix effects that may compromise the accuracy of direct measurements in complex samples. Consequently, the determination of etamsylate in samples with high concentrations of uric and ascorbic acids, such as urine, is not feasible due to substantial interferences from these compounds. To circumvent these limitations, the standard addition method was employed for the analysis of real samples. This approach effectively compensates for the sensitivity changes and potential fluctuations caused by the coexisting species. Furthermore, for samples with high concentrations of organic interferents, a preliminary dilution step or a pH adjustment of the supporting electrolyte is essential to further distinguish the etamsylate oxidation peak from overlapping signals and to reduce the background impact of the matrix to an acceptable level. In Figure 3, the relative changes in the ETM signal caused by the addition of interference are presented.

### 3.5. Analytical Performance

**Figure 4:**
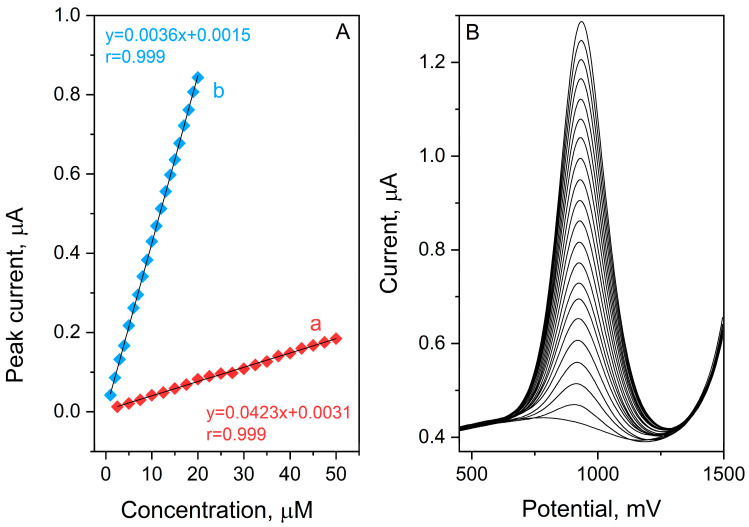
SW AdSV etamsylate calibration curves obtained for concentration ranges: 2.5–50 µmol L^−1^ (a) and 1–20 µmol L^−1^ (b) in 0.1 mol L^−1^ orthophosphoric acid (**A**). Corresponding voltammograms for calibration plot b (**B**).

The analytical capabilities of the BDDE for etamsylate quantification were evaluated by recording SWV voltammograms across a range of concentrations 1–20 µmol L^−1^ and 2.5–50 µmol L^−1^ in a 0.1 mol L^−1^ orthophosphoric acid (V) medium. Under the previously established optimal conditions, the sensor’s response was analyzed through two distinct calibration studies, utilizing ETM stock solution increments of 1 and 2.5 µmol L^−1^. To ensure statistical reliability, each measurement was performed in triplicate, with the resulting calibration curves based on the calculated mean peak currents (Figure 3). The validation parameters derived from these curves—including slope, intercept, linear dynamic range, and limit of detection (LOD)—are compiled in Table 1. The data in Figure 4 confirms a superior linear correlation between the ETM peak current and its concentration throughout the investigated ranges, evidenced by a correlation coefficient (*R*) of 0.999. The sensitivity of the method was further highlighted by a low detection limit of 0.10 µmol L^−1^, calculated using the following equation:

(4) $$LOD=\frac{3\sigma }{s}$$

where *σ* is a standard deviation from the intercept, and *s* is the slope of calibration curve.

Furthermore, the precision of the developed protocol was assessed through reproducibility tests. The Relative Standard Deviation (RSD) was calculated for seven successive measurements of a fixed ETM concentration (0.001 mol L^−1^), yielding a very good value of 3.17%. This low RSD underscores the exceptional stability and repeatability of the BDD electrode for etamsylate analysis.

To validate the practical utility of the proposed method, the BDD electrode was employed for the determination of etamsylate in both pharmaceutical tablets and spiked biological and environmental matrices. The BDD electrode was selected specifically for its superior electrochemical properties, including an exceptionally wide electrochemical potential window and high chemical stability, which are critical when analyzing complex samples. To address the signal enhancements and potential shifts observed in the interference studies the standard addition method was implemented (Point 2.5). This approach was essential to maintain analytical integrity by accounting for the specific matrix effects encountered on the BDD surface during the analysis of plasma of wastewater CRMs. The samples were prepared according to the procedure in Point 2.4. The analytical data, consolidated in Table 2, demonstrated robust recovery percentages between 95% and 102%. These results validate the high degree of sensitivity and practical efficacy of the BDD electrode for standard pharmaceutical quality assurance. Furthermore, the findings suggest that this electrochemical sensor is a viable tool for both clinical diagnostics and the detection of etamsylate residues in environmental monitoring applications.

## 4. Conclusions

The present study successfully demonstrates the high analytical potential of the Boron-Doped Diamond electrode for the sensitive determination of etamsylate. The electrochemical behaviour of etamsylate was also comprehensively investigated, resulting in the proposition of the ETM possible oxidation reaction on the BDDE. By utilizing Square Wave Voltammetry in a 0.1 mol L^−1^ orthophosphoric acid medium, a robust and reproducible analytical signal was achieved. The optimization of instrumental parameters led to a highly efficient procedure characterized by an excellent linear correlation (R^2^ = 0.999) and a low limit of detection (0.10 µmol L^−1^), which meets the requirements for both pharmaceutical and clinical monitoring. The BDD electrode itself exhibited remarkable stability and resistance to surface fouling, maintaining a precision of 3.17% expressed as RSD. In summary, the developed BDD-based method offers a fast, cost-effective, and reliable alternative to more complex and time-consuming chromatographic techniques. Its successful application in the analysis of pharmaceutical formulations and biological samples confirms its readiness for routine use in quality control and specialized medical diagnostics. We believe that demonstrating the high-performance capabilities of a simplified, robust system represents a significant contribution to the field of green and routine electroanalysis.

## Figures and Tables

**Table 1:** Analytical parameters obtained for the developed procedures for etamsylate determination.

Parameter	Linear Range	Slope	Intercept	R	LOD
Unit	µmol L^−1^	µA µmol^−1^	µA	-	µmol L^−1^
a	2.5–50 (0.66–13.16 mg L^−1^)	0.0036	0.0042 ± 0.00131	0.999	1.09 (0.287 mg L^−1^)
b	1–20 (0.26–5.26 mg L^−1^)	0.0423	0.0031 ± 0.00146	0.999	0.10 (0.026 mg L^−1^)

**Table 2:** Results of etamsylate determination in different samples.

Sample	ETM Declared, mg	ETM Found, mg	Relative Error, %
Tablets	500	493 ± 3	98.6
**Sample**	**ETM Added, µmol L^−1^**	**ETM Found, µmol L^−1^**	**Recovery, %**
Plasma 100 uL	1.0	0.99 ± 0.02	99.3
	2.0	1.95 ± 0.04	97.9
	3.0	3.03 ± 0.02	101.0
Waste Water SPS-WW1 300 uL	0.5	0.48 ± 0.01	95.4
	1.0	1.00 ± 0.03	100.0
	1.5	1.51 ± 0.02	100.5
Waste Water SPS-WW2 500 uL	0.5	0.49 ± 0.03	97.4
	1.0	1.02 ± 0.03	102.4
	1.5	1.49 ± 0.02	99.2

## Data Availability

The data sets used in this study are available from the corresponding author upon reasonable request.
